# Exploring STR sequencing for forensic DNA intelligence databasing using the Austrian National DNA Database as an example

**DOI:** 10.1007/s00414-021-02685-x

**Published:** 2021-08-26

**Authors:** Petra Hölzl-Müller, Martin Bodner, Burkhard Berger, Walther Parson

**Affiliations:** 1 Institute of Legal Medicine, Medical University of Innsbruck, Müllerstrasse 44, 6020 Innsbruck, Austria; 2 Forensic Science Program, The Pennsylvania State University, State College, PA USA

**Keywords:** Massively parallel sequencing, Sequence-based population data, Autosomal short tandem repeats, Allele frequencies, Reference samples

## Abstract

Here, we present the results from a population study that evaluated the performance of massively parallel sequencing (MPS) of short tandem repeats (STRs) with a particular focus on DNA intelligence databasing purposes. To meet this objective, 247 randomly selected reference samples, earlier being processed with conventional capillary electrophoretic (CE) STR sizing from the Austrian National DNA Database, were reanalyzed with the PowerSeq 46Y kit (Promega). This sample set provides MPS-based population data valid for the Austrian population to increase the body of sequence-based STR variation. The study addressed forensically relevant parameters, such as concordance and backward compatibility to extant amplicon-based genotypes, sequence-based stutter ratios, and relative marker performance. Of the 22 autosomal STR loci included in the PowerSeq 46GY panel, 99.98% of the allele calls were concordant between MPS and CE. Moreover, 25 new sequence variants from 15 markers were found in the Austrian dataset that are yet undescribed in the STRSeq online catalogue and were submitted for inclusion. Despite the high degree of concordance between MPS and CE derived genotypes, our results demonstrate the need for a harmonized allele nomenclature system that is equally applicable to both technologies, but at the same time can take advantage of the increased information content of MPS. This appears to be particularly important with regard to database applications in order to prevent false exclusions due to varying allele naming based on different analysis platforms and ensures backward compatibility.

**Supplementary Information:**

The online version contains supplementary material available at 10.1007/s00414-021-02685-x.

## Introduction

Throughout the past decades, short tandem repeat (STR) loci have become the most important genetic markers in forensics. They can be analyzed at a reasonable cost/time ratio and provide high enough statistical discrimination power to identify individuals in the majority of crime and human identification cases [1]. Traditionally, STRs are detected by PCR-generated amplicon sizing (also known as capillary electrophoresis-based methodologies, CE) and only relatively recently, massively parallel sequencing (MPS) approaches were introduced [2–5]. These studies revealed a number of benefits of MPS-based STR analysis over conventional CE methods, including, but not limited to, a larger number of loci and different types of loci that can be amplified in a single assay, an increased discrimination power by detecting isometric alleles (that share the same size but differ in sequence), and an increased success rate in deconvoluting complex mixtures [6]. Limitations that speak against an immediate implementation of MPS-based STR analysis approaches in a routine environment involve cost considerations, lack of harmonized sequence nomenclature, and lack of user-friendly software analysis tools, amongst others [7]. An important component, yet largely ignored, is the evaluation of current MPS-based STR typing in the routine environment of a forensic DNA databasing laboratory, although individual validation studies have touched on this topic (e.g., [8]). In addition to crime scene samples that are the primary focus of so far published studies, reference samples from suspects or convicted felons are required to be analyzed with the same technology in order to take full advantage of the methodological benefits.

Here, we evaluated the performance of an MPS-based STR-typing system consisting of the PowerSeq 46GY panel (Promega, Madison, USA) analyzed on a MiSeq FGx sequencer (Verogen, San Diego, USA) for DNA intelligence databasing purposes using a random subset of the Austrian National DNA Database as an example. The PowerSeq 46GY kit includes all loci required for national and international DNA intelligence databasing in the United States and Europe. We evaluated concordance and backward compatibility to extant CE-based genotypes, stutter display, and heterozygote balance and provide new population data to increase the body of STR variation that is currently being collected and catalogued in various environments (e.g., [9, 10]).

## Materials and methods

### Samples

All 248 buccal swab reference samples included in this study derived from the Austrian National DNA Database in accordance with the Austrian Data Protection regime. They were analyzed in line with Austrian legislation and with permission of the Austrian Federal Ministry of the Interior. Legal requirements with respect to sample storage (buccal swabs and/or extracted DNA) as well as the permission to go back to DNA extracts for re-testing are regulated by the Austrian Federal Security Police Act, which also contains specific legal provisions for scientific purposes to provide biometric data, including DNA data, in anonymized form to universities for research. The samples were randomly selected by executive authorities of the Austrian Federal Ministry of the Interior. The selection criteria were based on male sex, Austrian nationality, and birthplaces. The samples were made anonymous to the analyzing laboratory by using barcode information.

### DNA extraction

DNA was extracted from buccal swab samples using the Chelex 100 (Bio-Rad, Hercules, USA) method [11], and stored at − 20 °C for 1–13 years prior to DNA testing performed in this study. The selected samples were divided into three storage time groups as follows: group I: < 2 years; group II: 2 < 5 years, and group III: 5–13 years.

### DNA quantification

To determine the amount of genomic DNA, a real-time quantitative PCR (qPCR) assay targeting specific AluYb8 sequences was used [12]. A spiked in vitro mutagenized and cloned part of the human retinoblastoma susceptibility protein 1 (RB1) gene was co-amplified as an internal amplification positive control (pRB1-IPC) according to [13], updated in [14]. Calibration curve analyses covered a DNA input range from 169.5 fg to 10 ng per reaction and were performed in duplicates. The final reaction volume of 10 µL consisted of 5 µL TaqMan Fast Universal PCR mix (Thermo Fisher Scientific [TFS], Waltham, USA), 3 µL primer probe premix (made in-house), and 2 µL extracted DNA. The amplification was carried out on an Applied Biosystems 7500 Fast Real-Time PCR instrument (TFS) applying 95 °C for 20 s, 40 cycles of 95 °C for 3 s, and 60 °C for 30 s. Data analysis was carried out with the HID Real-Time PCR Software v 2.3 (TFS). Kinetic information for the pRB1-IPC system yielded no indication of inhibition during DNA amplification.

### Capillary electrophoresis

STR analysis was performed using the AmpFlSTR NGM SElect Express kit (TFS) [15] and the PowerPlex 16 System (Promega) [16] on all samples, resulting in a total of 23 autosomal STR (aSTR) loci plus amelogenin. As SE33 is not included in the PowerSeq 46GY panel, only length-based SE33 data could be considered. All remaining 22 aSTR markers as well as amelogenin are also included in the PowerSeq 46GY panel enabling a comparative view of the results. Amplifications were performed on ABI GeneAmp 9700 thermal cyclers (TFS) following the recommended protocols [15, 16]. PCR products were separated and detected using an Applied Biosystems Prism 3500XL Genetic Analyzer (TFS).

### MPS workflow

The PowerSeq 46GY kit (Promega) was used to co-amplify 22 aSTRs (D1S1656, TPOX, D2S1338, D2S441, D3S1358, FGA, D5S818, CSF1PO, D7S820, D8S1179, D10S1248, TH01, vWA, D12S391, D13S317, Penta E, D16S539, D18S51, D19S433, D21S11, Penta D, and D22S1045), 23 Y-STRs (data not shown), and amelogenin. This extended STR panel aims to target forensic markers to comply with the European Standard Set (ESS) [17, 18] and the Combined DNA Index System (CODIS) recommendations [19–21]. Amplification, purification, library preparation, normalization, quantification, pooling, and sequencing were performed according to the manufacturer’s recommendations [22–24].

The 248 DNA samples were processed as five library batches of 4 × 48 and 1 × 56 samples (Table 1). Each library batch included a positive and a negative amplification control, resulting in 50 (4 ×) or 58 (1 ×) samples (incl. controls) that were assembled into one sequencing run. After sequencing and data analysis, one sample was excluded due to contamination during manual library preparation. Therefore, only data of the remaining 247 samples were further considered.

**Table 1:** Run and quality metrics information for five sequencing runs. Sequencing was performed using the PowerSeq 46GY kit (Promega, USA) on a MiSeq FGx instument (Verogen, USA) according to the manufacturer’s recommendations

	Cluster density (K/mm^2^) (*1000–1200 K/mm*^*2*^)	Cluster passing filter (PF; %)	Phasing (%)	Pre-phasing (%)	Total no. of reads	Total no. of reads PF	% ≥ Q30 (> *75%*)	Number of samples/run (including two controls)
Run 1	495 ± 14	97.29 ± 0.18	0.107	0.134	9,739,105	9,474,979	90.0	50
Run 2	1466 ± 22	82.47 ± 1.45	0.124	0.116	27,486,348	22,667,346	80.8	50
Run 3	1141 ± 25	89.26 ± 0.58	0.127	0.121	21,667,218	19,339,568	81.8	50
Run 4	1202 ± 27	87.29 ± 1.39	0.114	0.125	22,705,688	19,821,280	79.5	50
Run 5	920 ± 20	89.92 ± 1.34	0.113	0.138	17,228,100	15,489,251	84.0	58

#### Amplification of STR fragments

All samples were diluted accordingly in molecular grade water to amplify 0.5 ng of template DNA according to [22]. Multiplex PCR was performed targeting 0.5 ng DNA using the PowerSeq 46GY kit [22] on an Applied Biosystems GeneAmp 9700 thermal cycler (TFS) according to the manufacturer’s recommendations [23]. Each amplification reaction was treated with 5 µL proteinase K solution [504 µg/mL] (Roche Diagnostics, Mannheim, Germany) and purified using AMPure XP beads (Beckman Coulter, CA, USA) following [22]. The concentrations of amplification products were estimated spectrophotometrically prior to library preparation by measuring the absorbance at 260 nm according to [25] and as recommended in [23] with a NanoDrop ND-1000 spectrophotometer and analyzed with software version 3.8.1 (both Peqlab Biotechnologie GmbH, Erlangen, Germany).

#### Library preparation and sequencing

End-repair, A-tailing, adaptor ligation, initial, and second purification were performed using the TruSeq DNA PCR-Free HT Library Prep Kit (Illumina, San Diego, USA; [24]) according to the manufacturer’s recommendations [22, 23], with the exception that supplied sample purification beads were replaced by AMPure XP beads (Beckman Coulter, CA, USA). To ensure balanced pooling, each library sample was quantified in duplicate by means of qPCR using the KAPA SYBR FAST Universal qPCR Kit (Roche) following [26] on an Applied Biosystems 7500 Fast Real-Time PCR instrument. Data analysis was performed using the HID Real-Time PCR Software v 2.3. Based on qPCR results, samples were diluted and normalized to 4 nM and equally pooled according to [22]. Sequencing was performed on the MiSeq FGx instrument (Verogen, [27]) using a 500 cycles MiSeq Reagent Kit v2 for 2 × 250 paired-end sequencing (Illumina, [28]) according to the manufacturer’s recommendations. The final library concentration was 12 pM with approx. 6.6% spiked PhiX control library (Illumina) following [22].

### Data analysis

#### Capillary electrophoresis data

Size-based analysis of STR fragments was conducted using the GeneMapper ID-X software, version 1.2 (TFS) by applying in-house validated dye thresholds: blue — 50 relative fluorescence units (RFU), green — 80 RFU, yellow — 100 RFU, and red — 100 RFU.

#### MPS data

Sequencing results were monitored using the Sequencing Analysis Viewer software (Illumina; [29]) to review relevant quality metrics. Compressed FASTQ files were manually extracted for data analysis using the open-source STRait Razor v2s software tool [30, 31]. Sequences were aligned to human genome assembly GRCh38 and genomic coordinates for STR markers were determined by post processing of the mpileup output from SAMtools [32] as described in [30, 31]. STR genotypes were analyzed by applying an analytical threshold (AT) of 50 reads, referring to [33]. Alleles were called above the in-house defined interpretation threshold (IT) of 500 or 100 reads for homozygous or heterozygous genotypes, respectively. Sequence-based allele frequencies are shown in Table S1 considering all available sequence information.

#### MPS stutter ratios

Stutter analysis was restricted to a subset of 50 samples (app. 20% of the entire sample set) selected according to the total number of reads that was calculated by summarizing the intensity (read count) of a given STR profile for all 22 aSTRs. Stutter sequences were determined as one repeat unit smaller than the parental allele and calculated by dividing the intensity of stutter sequence by the intensity of the corresponding allele. The selected samples were divided in two equally sized groups comprising low (category I) and high performing samples (category II), respectively. Selection criteria were established to investigate the effect of sample performance (total number of reads per genotype) on the formation of stutter height and defined as follows: category I comprised only samples that fell below 63,500 reads, while category II included solely samples above 199,000 reads (Table S2).

#### Statistical analysis

Microsoft Excel workbooks, IBM SPSS software, version 24 [34], and GraphPad Prism, version 8 for Windows [35], were applied.

Forensic and population genetic parameters, including allele frequency, observed and expected heterozygosity, expected homozygosity, power of exclusion, power of discrimination, matching probability, typical paternity index, and exact Hardy–Weinberg equilibrium (HWE) tests for the population, were calculated from CE data using in-house software according to formulae listed in Table S3 and the STRAF software package [36].

## Results and discussion

This population study comprised samples that were collected between August 2005 and July 2017. Size-based STR genotypes were generated in the course of routine forensic practice when the samples entered the laboratory using conventional CE.

### MPS run parameters

To evaluate the data quality of each sequencing run, we extracted the provided quality metrics, e.g., cluster density, reads passing filter, Q30 scores, and data output. Table 1 shows the attained run parameters (recommended values in brackets): cluster density (1,000–1,200 K/mm^2^), cluster passing filter, phasing, pre-phasing, total number of reads, total number of reads passing filter, % ≥ Q30 (> 75%), and the number of samples per run.

One of the runs (run 3) generated optimal cluster density according to the manufacturer’s recommendation [37] (Table 1), whereas cluster densities for runs 1 and 5 as well as for runs 2 and 4 were diagnosed as under- and overclustered, respectively. Overclustering potentially introduces analytical problems, which might lead to poor run performance and decreases Q30 scores along with lowered data output. In contrary, underclustering does not inevitably harmfully affect data quality but predominantly lowers data output. *Q*-scores, also known as Phred quality scores, are commonly used metrics of base calling accuracy and to communicate very small error probabilities. For example, a *Q*-score of 30 assigned to a base call is identical to the probability of an incorrect base call 1 in 1,000 times, i.e., base call accuracy is 99.9% [38, 39]. In the current study, all sequencing runs exceeded the recommended Q30 score of > 75% (Table 1) indicating reliable base calling (mean: 83.2%; standard deviation (SD): 4.1%). In this study, we considered only MPS-based aSTR genotype calls that met the in-house defined interpretation threshold (see “MPS data” section).

### Concordance

Concordance was evaluated for the 22 aSTR markers shared between the PowerSeq 46GY kit and the applied CE kits (AmpFlSTR NGM SElect Express Kit and PowerPlex 16 System) by comparing length-based allele calls recalculated from MPS results and those derived from CE. The only discordance between both technologies was caused by two allelic drop-out events: in one case, drop-out of the longer allele was found at D2S1338 (CE: 18/28; MPS: 18; 1750 reads) and in the other case, the shorter allele at Penta E (CE: 8/11; MPS: 11; 3029 reads) dropped out. Full concordance was observed between the applied CE kits for the eight autosomal STR loci included in both multiplex assays.

All results for the positive amplification controls were concordant to the known information, except a partial but otherwise correct profile of the positive control in run 2. Repeat-region sequence information was concordant for all typed positive amplification control alleles. No negative amplification control yielded a detectable signal, with the exception of one allelic drop-in in run 4 (TPOX: allele 11; 373 reads).

Allele concordance between MPS and CE was 99.98% (10,866 out of 10,868 alleles in total) and locus concordance amounted to 99.96% (5,432 out of a total of 5,434 STR loci; Table S4). These findings are comparable to earlier reported concordance studies [40–42]. Furthermore, concordance was similar to that observed between commonly used CE-STR kits [43, 44] indicating that the results obtained with the PowerSeq 46GY panel are highly compatible with those obtained by standard STR-typing technologies.

### PowerSeq 46GY kit performance

#### DNA quantity and storage period

The mean DNA quantity of the 247 samples was 7.29 ng/µL (SD: 5.20, median: 6.23) with a minimum of 0.71 ng/µL and a maximum of 33.04 ng/µL (Table S5). The mean storage period for DNA samples used within the current study was 2.96 years (SD: 1.90; median: 3.12) and varied between 1 and 13 years (Table S5). As there were no changes in sampling and DNA extraction over the entire time period, the latter is the only remaining variable that could influence the DNA quantity and quality. As shown in Figure S1, storage time did not affect sample performance and was comparable for the majority of samples included in each storage time group (Figure S1). Although relative marker performance decreased slightly with increasing amplicon length, we did not observe differences between storage time groups I–III (Figure S2). The decline in relative marker performance, in relation to amplicon size, did not adversely affect downstream data analysis.

#### Relative marker performance

**Fig. 1:**
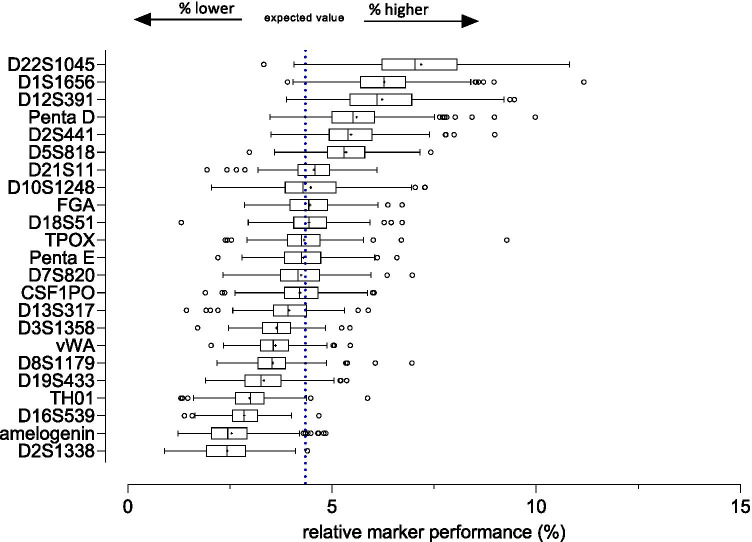
Relative marker performance showing box-whisker plots of the PowerSeq 46GY kit comprising 23 markers (including amelogenin). The expected value (dotted line, mean shown as “ + ”) marks the proportion of reads for a given marker, assuming 100% equally performing markers included in the PowerSeq 46GY panel (*n* = 23; expected value = 4.35%). The standard deviation for the mean relative marker performance was 1.23%

Relative marker performance was evaluated (for each sample and STR locus) by dividing the intensity of marker reads by the total number of reads for all markers of a single STR profile. Assuming equally performing STR markers, the expected relative marker performance value for each of the 23 markers (incl. amelogenin) of the PowerSeq 46GY panel would equal 4.35% of all reads. The effective mean relative marker performance values were found to lie close to the expected value and were comparable for the majority of markers included in the present MPS panel, except for D1S1656, D2S1338, TH01, D12S391, D16S539, Penta D, D22S1045, and amelogenin (Fig. 1). The average performances of the latter eight markers were outside the well-balanced range, which we defined as mean relative marker performance + / − one SD_(marker mean)_ (SD_[marker mean]_: 1.23%; Table S5). These results indicated that the PowerSeq 46GY prototype library kit consists of a more balanced marker set compared to other prototype/early access MPS-based STR multiplexes, when only aSTRs are considered [45].

#### Heterozygote balance

Heterozygote balance (HB) is expressed (for each sample and STR locus) as ratio between the minor and the major allele intensity for heterozygote genotypes. On average, all markers showed HB ratios ≥ 0.80, except D12S391 (mean: 0.79; SD: 0.13), D19S433 (0.78; 0.14), Penta E (0.77; 0.15), and D2S1338 (0.70; 0.19) (Figure S3, Table S4). In seven samples (0.13% of the sample set), we observed highly imbalanced genotype calls (HB ratios ≤ 0.30) at D2S1338 (5 ×), D19S433 (1 ×), and D21S11 (1 ×) (Figure S3, Table S4). Generally speaking, HB ratios were similar to those obtained with CE-based STR kits [46, 47] or reported in earlier MPS studies [48, 49]. D2S1338 was found to be most susceptible to heterozygote imbalance, which has been described by [49] before. Since, high imbalances potentially lead to marker drop-out, further optimization steps, especially for D2S1338, are required as already indicated by [49]. We note that known imbalances at D22S1045 [2, 50–52] and D5S818 [51, 53–55] were not observed for genotypes amplified with the PowerSeq 46GY library kit. In one sample, heterozygote imbalance was found at D5S818 after CE analysis but not with MPS (typed alleles — CE: 10/11, HB: 20%; MPS: 10/11, HB: 72.1%). Sequence analysis of the latter sample unveiled the presence of an A > G transition (rs182073376), which was located 36 nucleotides upstream the repeat region of D5S818 [56] and was found only once within our dataset. To understand the reason for this imbalance, we examined the SNP location with respect to the previously published PowerPlex 16 (Promega) primer sequences [57]. Indeed, the observed A > G transition was located ten nucleotides upstream of the 3′ end within the forward primer’s binding site, which apparently decreased thermal stability of the primer-template complex and, therefore, reduced PCR performance (Figure S4) [58, 59].

#### Stutter ratios

Stutter products are commonly known artifacts in STR typing that are caused by strand slippage of the DNA polymerase during the extension phase of PCR. Strand slippage results in the addition or deletion of typically one repeat unit in the nascent DNA strand [60]. Besides of rarely seen stutter products in *n* + 4 and *n* − 8 positions, the most prominent observed stutters are one repeat unit shorter, i.e., *n* − 4 for tetranucleotide STR markers.

Note, by applying MPS for STR analysis, each repeating motif may result in the formation of a, i.e., *n* − 4, stutter product and is therefore multivariate [61]. Furthermore, the formation of stutter seems to be also influenced by adjacent nucleotides [61]. As only single source samples were analyzed, we decided to reduce the complexity of sequence-based stutter analysis by considering stutter products as “defined by length” (univariate; recalculated from MPS results) instead of as “defined by sequence” (multivariate). As expected, stutter ratios increased with the growing number of repeat units (allele size) [60, 62]. Locus-specific stutter analysis showed that stutter ratios for integer alleles were higher than those for intermediate alleles (Figure S5), which is in line with earlier reports [61]. We found no evidence of increased stochastic variation of stutter height for samples belonging to category I (samples with reads < 63,500; Table S2), which is in contrast to the findings described by [62].

Stutter ratios were found to be similar for category I and category II samples (Figure S5; Table 2). The majority of STRs included in the PowerSeq 46GY panel showed mean stutter ratios ranging from 10 to 15% for both categories (Table 2). Stutters appeared to be generally higher for MPS-based STR genotyping than for CE-based kits [46, 47, 63] (Table 2), which is in line with earlier reports [45, 64–66]. Rarely, we observed stutter values exceeding 20% (Table 2). However, as D22S1045 consists of a trinucleotide repeat, which is more prone to stutter artifacts, higher stutter values were expected and also known from earlier studies [44, 45, 67].

**Table 2:** Global stutter analysis was performed for 22 forensic markers on a subset of the Austrian population sample (*n* = 50). Samples were selected according to the total number of reads and divided into two categories (selection criteria: category I: ≤ 63,500 reads; category II: ≥ 199,000 reads). Bold numbers denote stutter values exceeding 20%. In general, mean stutter values were comparable to CE-based stutter heights

	Global stutter analysis — category I							Global stutter analysis — category II						
Marker	*n*_[total]_	Mean	SD	Median	Min	Max	Stutter > 20% (*n*)	*n*_[total]_	Mean	SD	Median	Min	Max	Stutter > 20% (*n*)
D22S1045	30	15.2	3.9	14.9	6.7	**28.8**	2	39	13.4	4.8	14.0	6.4	**26.0**	2
D18S51	40	13.1	3.4	13.4	8.5	**23.5**	2	44	12.2	2.8	12.0	7.8	18.8	
D1S1656	46	14.0	3.6	13.1	9.1	**21.6**	5	45	14.3	3.9	13.0	8.3	**22.3**	4
D2S1338	43	13.3	3.6	12.7	7.1	**21.4**	3	46	12.9	3.2	12.2	8.0	**21.0**	1
D19S433	37	12.9	2.7	12.8	8.4	**21.3**	1	36	14.1	2.4	13.4	10.1	**20.4**	2
D10S1248	33	13.1	2.8	11.9	9.9	**21.3**	2	33	13.7	3.1	12.6	9.3	**22.9**	1
D12S391	48	12.2	3.5	12.3	3.3	19.5		46	11.8	4.0	12.2	3.7	19.0	
FGA	38	12.0	2.8	12.0	7.1	18.7		36	12.4	2.5	12.5	7.8	17.0	
D3S1358	46	12.0	2.0	12.0	8.7	18.6		43	12.7	2.2	12.8	8.7	17.3	
vWA	33	12.2	2.8	12.1	7.7	18.3		36	12.7	2.2	12.2	7.0	18.1	
D8S1179	40	10.9	2.2	10.9	6.8	16.9		40	10.5	2.1	10.5	5.8	16.5	
D16S539	39	10.5	2.6	10.5	6.3	15.7		38	10.7	2.4	10.9	5.7	15.3	
D13S317	34	7.7	2.4	7.4	3.5	14.1		44	6.7	2.5	7.1	2.9	12.5	
D21S11	40	9.7	2.0	9.6	6.2	14.1		45	9.4	1.5	9.4	4.4	12.9	
CSF1PO	31	8.0	1.8	7.6	5.2	13.4		27	7.6	1.3	7.4	5.1	10.0	
D7S820	40	8.8	2.0	8.7	4.7	13.2		39	8.0	2.1	7.8	3.9	12.1	
Penta E	27	7.0	1.8	6.4	5.0	11.8		43	5.7	2.3	5.8	0.9	11.3	
TPOX	29	5.7	1.9	5.4	3.3	11.7		40	5.1	1.2	5.1	3.4	8.2	
D5S818	44	8.5	1.7	8.7	4.8	11.6		43	9.3	1.9	9.0	5.7	14.5	
D2S441	35	6.0	2.1	5.7	2.4	9.4		39	6.4	2.2	6.4	3.2	10.2	
TH01	13	6.8	1.4	6.5	4.6	9.1		39	4.3	1.4	3.8	2.0	7.6	
Penta D	1	5.2	NA	5.2	5.2	5.2		39	1.9	0.7	1.9	0.6	3.8	

### Sequence variation

As expected, MPS genotyping increased the detection of genetic variation compared to the length-based procedure via CE. For the following 19 of 22 aSTRs, we observed sequence variation not detectable with CE technology: D1S1656, TPOX, D2S1338, D2S441, D3S1358, FGA, D5S818, CSF1PO, D7S820, D8S1179, TH01, vWA, D12S391, D13S317, D16S539, D18S51, D19S433, D21S11, and Penta D (Table 3, Figure S6). Details on sequence variation can be found in Table S6 that used the updated Forensic STR Sequence Structure Guide v5 [56], available on the STRidER (https://strider.online/) website [68], as template.

**Table 3:** Overview of sequence variation observed within the Austrian population using the PowerSeq 46GY kit: As expected, MPS techniques revealed increased genetic variation compared to length-based technologies. To characterize the location of sequence variation, we used repeat and flanking region definitions reported in the updated Forensic STR Sequence Structure Guide v5 (Phillips 2018). Due to the lack of a harmonized MPS allele nomenclature that would also define flanking region lengths, we considered the fully available sequence strings up- and downstream from the repeat region as flanking regions. Size-based STR analysis was performed using the AmpFlSTR NGM SElect Express kit (Thermo Fisher Scientific, USA) and the PowerPlex16 kit (Promega, USA)

	Number of different alleles observed		Increase in sequence variation		Region of sequence variation	
Marker	Length-based	Sequence-based	No. of alleles	x-fold↑	Repeat	Flanking
D12S391	16	53	37	3.3	◊	
D2S1338	11	33	22	3.0	◊	•
D21S11	14	36	22	2.6	◊	
D3S1358	7	20	13	2.9	◊	
vWA	7	19	12	2.7	◊	•
D7S820	8	20	12	2.5		•
D5S818	7	18	11	2.6		•
D8S1179	10	19	9	1.9	◊	
D1S1656	16	25	9	1.6	◊	•
D13S317	9	16	7	1.8		•
Penta D	12	18	6	1.5		•
D16S539	9	14	5	1.6		•
D2S441	11	15	4	1.4	◊	•
FGA	15	18	3	1.2	◊	
D19S433	15	18	3	1.2	◊	•
TPOX	6	8	2	1.3		•
D18S51	14	16	2	1.1	◊	
TH01	7	8	1	1.1	◊	•
CSF1PO	8	9	1	1.1	◊	•
D10S1248	9	9	-	-	-	-
Penta E	16	16	-	-	-	-
D22S1045	9	9	-	-	-	-

The loci D12S391 (3.3-fold), D2S1338 (3.0-fold), and D21S11 (2.6-fold) showed the most pronounced increase in the number of distinguishable alleles by sequence-based analysis (Figures S7a–c), which have already been found highly variable in earlier studies [6, 40–42, 49, 69, 70]. Interestingly, we observed sequence variation at TPOX (Table S1, Table S6, Figure S6). This differs from numerous earlier studies [40–42, 49, 70–72] but is consistent with three reports [6, 66, 69] that recorded sequence variation at TPOX. Our results were confirming those of [6, 66] who reported flanking region SNPs at TPOX, whereas [69] only observed sequence variation at TPOX within the repeat region. In line with earlier studies, no additional sequence variation was found for D10S1248 [40, 41, 49, 66, 71], Penta E [40], and D22S1045 [40, 42, 66] (Table 3).

#### Isometric alleles

Sequence-based analysis revealed an increase of heterozygosity relative to CE-based genotyping due to isometric alleles (alleles of identical size but different sequence). Using MPS, 181 of the 1075 (16.8%) homozygous allele pairs were unveiled as isometric heterozygotes at 13 aSTRs: D5S818 (34 ×), D3S1358 (25 ×), D13S317 (23 ×), D21S11 (16 ×), D7S820 (15 ×), D8S1179 (14 ×), D16S539 (14 ×), vWA (11 ×), D2S1338 (9 ×), D2S441 (8 ×), D12S391 (6 ×), D1S1656 (4 ×), and TPOX (2 ×) (Table 4). Our findings for increased heterozygosity were in line with an earlier report [42], except for TPOX. For example, Gettings et al. (2018) [69] observed a minimal increase in heterozygosity (< 1%) at TPOX, while Silva et al. (2020) [49] did not observe sequence variation at TPOX, D7S820, and D13S317. In two samples, MPS was able to identify isometric heterozygous genotypes at TPOX, allele 8 (repeat structure variants [AATG]8 vs. [AATG]8 containing a G > T transversion located in the flanking region [rs149212737]).

**Table 4:** Overview of STR markers showing the total number of observed alleles using two different STR analysis technologies. MPS increased heterozygosity by identifying 181 homozygous genotypes as isometric heterozygotes. Isometric alleles, also known as isoalleles, are alleles of identical length but different internal sequence

	Total number of alleles obtained			Heterozygosity	
Marker	Length	Sequence	Isoalleles (*n*)	Length-based	Sequence-based
D5S818	434	468	34	0.88	0.95
D3S1358	429	454	25	0.87	0.92
D13S317	432	455	23	0.87	0.92
D21S11	449	465	16	0.91	0.94
D7S820	449	464	15	0.91	0.94
D8S1179	454	468	14	0.92	0.95
D16S539	439	453	14	0.89	0.92
vWA	448	459	11	0.91	0.93
D2S1338	455	464	9	0.92	0.94
D2S441	431	439	8	0.87	0.89
D12S391	473	479	6	0.96	0.97
D1S1656	468	472	4	0.95	0.96
TPOX	417	419	2	0.84	0.85
FGA	455	455	-	0.92	0.92
CSF1PO	425	425	-	0.86	0.86
D10S1248	435	435	-	0.88	0.88
TH01	432	432	-	0.87	0.87
Penta E	465	465	-	0.94	0.94
D18S51	463	463	-	0.94	0.94
D19S433	449	449	-	0.91	0.91
Penta D	455	455	-	0.92	0.92
D22S1045	433	433	-	0.88	0.88

#### Benefits and limitations of STR sequence data in DNA intelligence databasing

The primary intention of this study was to characterize the full sequence information of STRs with respect to forensic DNA intelligence databasing. Despite a high degree of concordance between MPS and conventional CE methodologies, we observed sequence variation that could potentially cause differences between the apparent CE length and allele calls based on counting repeat units in the MPS data as applied below. Furthermore, and as described earlier, sequence variation adjacent to the repeat region is known to affect allele nomenclature based on repeat unit counting [73]. In addition, Bodner and Parson (2020) [9] reported that SNP- and insertion/deletion-caused discrepancies between MPS and CE allele sizes were also a main reason of errors and discrepancies detected during STRidER quality control of MPS datasets.

Instances of flanking region deletions were observed at D2S441, D19S433, and Penta D, respectively (Table S6). At D2S441, we identified a single [T/-] deletion (rs888232687) of the first base downstream of the repeat block (CE: 9.3; MPS: 10) [56]. At D19S433, alignment revealed a single [CT/-] deletion (rs745607776) located in the 5′ flanking region (CE: 14.2; MPS: 15) that was previously described [42, 45, 56]. At Penta D, we identified a 13-nucleotide [AAGAAAGAAAAAA/-] deletion (rs1190908807) that results in the length-based allele 2.2 previously reported by [42, 56, 69]. Additionally, we observed a [A/-] deletion (rs536566765) located in the 3′ flanking region of Penta D (CE: 13.3; MPS: 14) that could cause discordance between MPS and CE as previously reported by [71, 74]. Considering sequence information, the two aforementioned samples revealed five and 14 full repeat units, respectively (Table S6). We note that all these described deletions would have caused seemingly shorter amplicons resulting in discrepant allele size estimations by mimicking intermediate alleles with CE-based detection methods (Table S6).

In addition, at D19S433, we identified 55 intermediate alleles (CE: X.2; representing 11.1% of all D19S433 alleles) that contained a well-characterized [TC/-] deletion (rs147936416; Table S6) [56]. This particular polymorphism is located inside the repeat region of D19S433, and spans the border of a counted repeat unit and an uncounted nucleotide block (for more information, see [56, 73]). This might lead to different micro-variant allele designation depending on the technology and counting method used. For example, an allele called 12.2 based on CE would result in an allele 12.3 based on repeat unit counting from MPS results (Table S6). Of note, alignment of these micro-variant alleles currently also differs in catalogues [10, 56]. As any kind of insertion/deletion can impact concordance between MPS and CE, it is inevitable to come up with a straight forward nomenclature system that maintains backward compatibility to the size-based STR profiles stored in national DNA databases. Based on this, it is important to collect and describe as many sequence variants as possible to increase sequence-based genotype accuracy [71].

### Allele variation and frequencies, forensic and population genetic parameters, STRidER quality control, and databasing

All STR alleles found in the CE dataset have, in addition to confirmation by MPS, previously been reported on STRidER [68] or pop.STR [75] at similar frequencies, or were rare variants listed in the NIST STRBase [76]. Results for forensic and population genetic parameters revealed generally high diversity for all loci and thus their suitability for forensic applications. For all parameters, TPOX was the least and SE33 the most diverse locus. All loci met HWE expectations with *p*-values for deviations above 0.05 (Table S3). This is similar to other datasets [40, 42, 77]. Resulting STR allele frequencies, forensic and population genetic parameters calculated from the 245 complete CE genotypes are available from Table S3. The CE dataset was submitted to STRidER [68], including only complete genotypes according to its databasing requirements [9]. Two samples yielded three allelic genotype calls at SE33 in CE, using the AmpFlSTR NGM SElect Express kit [15], and were therefore excluded from the dataset submitted to STRidER. The 245 CE-based and 247 MPS-based datasets passed STRidER quality control [9] and were assigned accession numbers STR000249 (CE) and STR000337 (MPS). The Austrian allele frequencies will augment the quality-checked data available to the community from the STRidER online allele frequency database [68]. MPS-based allele frequencies of 22 autosomal STRs are shown in Table S1 and will enable statistical calculations from sequenced DNA profiles for the Austrian population. Note that these allele frequencies (Table S1) should be considered preliminary due to the lack of a generally recognized allele nomenclature and recommended sequence ranges. They will be presented on STRidER once these prerequisites for forensic population databasing are agreed on [78].

Data analysis revealed 25 novel sequence variants that have been undescribed in the STRSeq online catalogue so far [10] at loci D1S1656 (3 ×), TPOX (1 ×), D2S441 (1 ×), D3S1358 (2 ×), FGA (1 ×), CSF1PO (1 ×), D7S820 (1 ×), TH01 (1 ×), D12S391 (2 ×), D13S317 (1 ×), D16S539 (1 ×), D18S51 (1 ×), D19S433 (2 ×), D21S11 (3 ×), and Penta D (4 ×). They were submitted for inclusion in STRSeq [10] (Table S1, Table S7; BioProject accession: PRJNA380345; aSTR sequence data: Nucleotide (Genomic DNA) – 1386; date of database query: 28/01/2021).

Of note, this first genotype set where complete allelic data from both CE and MPS analysis were submitted to STRidER enabled direct comparison of the degree of identity of the resulting genotypes. From the perspective of a posteriori quality control [9], the findings further contribute to genuine assessment of applicability and pitfalls of “simply” using length-based allele calls recalculated from MPS results as pseudo-CE alleles for databasing.

## Conclusions

This study investigates the performance of MPS for forensic STR intelligence databasing purposes taking a set of 247 randomly picked male individuals from the Austrian National DNA Database as example. The PowerSeq 46GY kit analyzed on the MiSeq FGx system resulted in reliable base calling irrespective of cluster density variations. The investigated aSTR genotypes were highly concordant compared to conventional CE sizing approaches with some exceptions that were also observed in earlier studies [42, 45, 71]. However, differences between CE- and MPS-based results can lead to false exclusions when only length-based alleles, recalculated from MPS results by repeat unit counting, are considered as pseudo-CE alleles and used for DNA intelligence databasing purposes. This would have been the case for two samples due to one mismatch each at D2S441 and D19S433 if our dataset had been imported into the Austrian National DNA Database in this way. From a technical point of view, this reinforces the requirement to use an error-tolerant search algorithm when comparing/searching STR genotypes in intelligence databases that already need to deal with discrepancies between different CE-based STR typing kits.

Sequence-based stutter analysis showed comparable ratios to CE for both low and high performing MPS samples with a small tendency for higher stutter in MPS data.

As expected, we observed substantial sequence variation located within the repeat motif and the flanking region for the majority of STR markers. Only few loci showed no gain in discrimination when comparing sequence-based with length-based allele calls. In general, our results were comparable to previously published population studies [6, 41, 42, 49, 69, 70, 72].

## Supplementary Information

Below is the link to the electronic supplementary material.

Supplementary file1 (PDF 132 KB)

Supplementary file2 (PDF 134 KB)

Supplementary file3 (PDF 96 KB)

Supplementary file4 (PDF 175 KB)

Supplementary file5 (PDF 174 KB)

Supplementary file6 (PDF 138 KB)

Supplementary file7 (PDF 122 KB)

Supplementary file8 (XLSX 1015 KB)

## Declarations

### Disclaimer

This publication reflects the views only of the authors, and the European Commission cannot be held responsible for any use, which may be made of the information contained therein.
