# Supplementary material for: Exploring STR sequencing for forensic DNA intelligence databasing using the Austrian National DNA Database as an example
Source: Int J Legal Med. 2021 Aug 26;135(6):2235–46. doi: 10.1007/s00414-021-02685-x (PMC8523457; doi:10.1007/s00414-021-02685-x)
Supplement: Supplementary file 1 — Supplementary file1 (PDF 132 KB) [file 414_2021_2685_MOESM1_ESM.pdf]

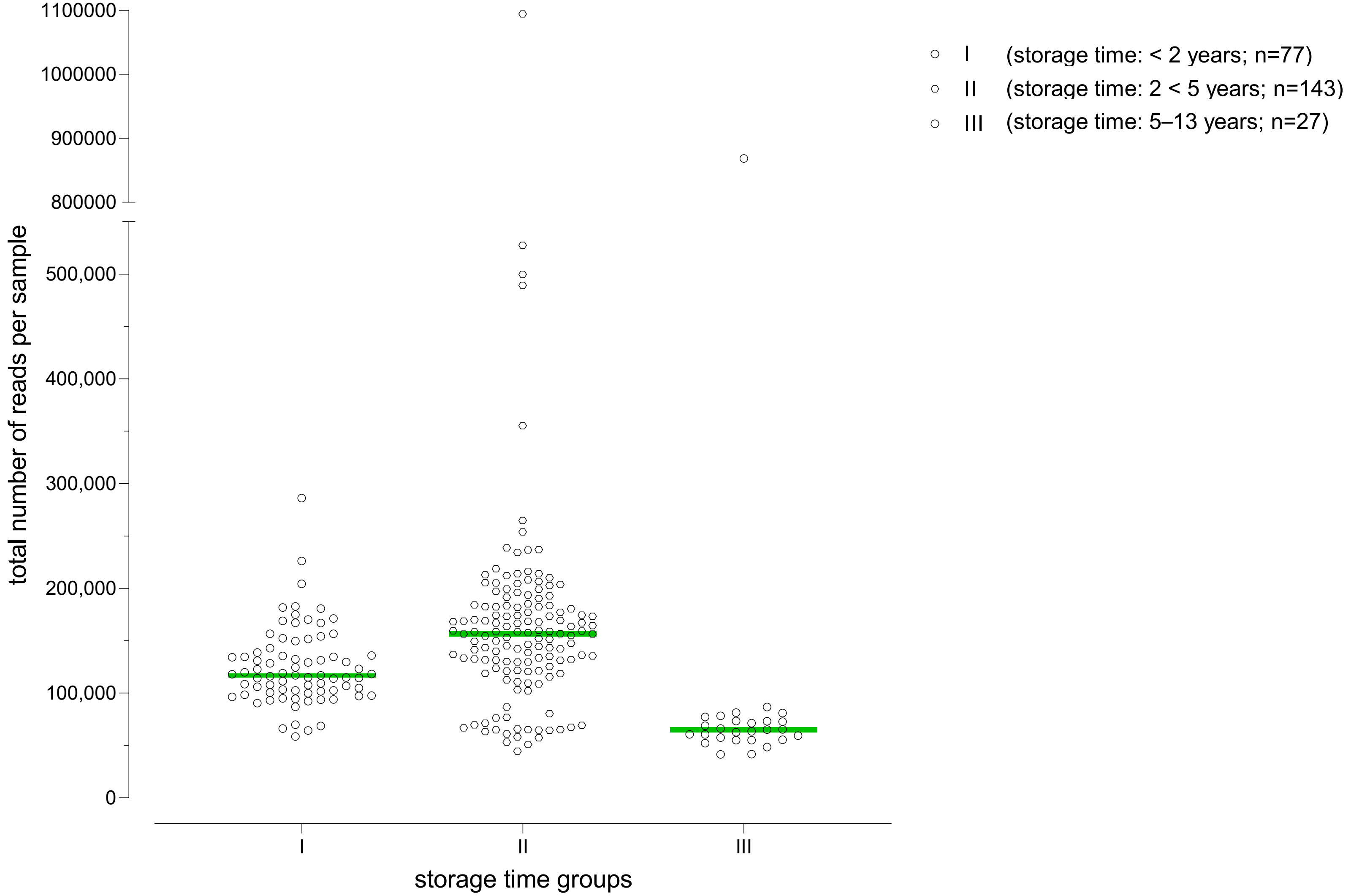

Fig. S1 Sample performance obtained for three storage time groups (green line at median). The absolute sample performance (each sample is represented as circle) was plotted against storage time prior to MPS analysis. In general, the total number of reads per sample was comparable for the majority of specimens included in each storage time group. Overall, sample performance was almost halved for samples falling in storage time group III (median: 65092 reads) compared to those included in group I (median: 116903 reads). The decrease in sample performance did not adversely affect downstream data analysis.

Hölzl-Müller P., Bodner M., Berger B., and Parson W. "Exploring STR Sequencing for Forensic DNA Intelligence Databasing using the Austrian National DNA Database as an Example"
