# Supplementary material for: Exploring STR sequencing for forensic DNA intelligence databasing using the Austrian National DNA Database as an example
Source: Int J Legal Med. 2021 Aug 26;135(6):2235–46. doi: 10.1007/s00414-021-02685-x (PMC8523457; doi:10.1007/s00414-021-02685-x)
Supplement: Supplementary file 2 — Supplementary file2 (PDF 134 KB) [file 414_2021_2685_MOESM2_ESM.pdf]

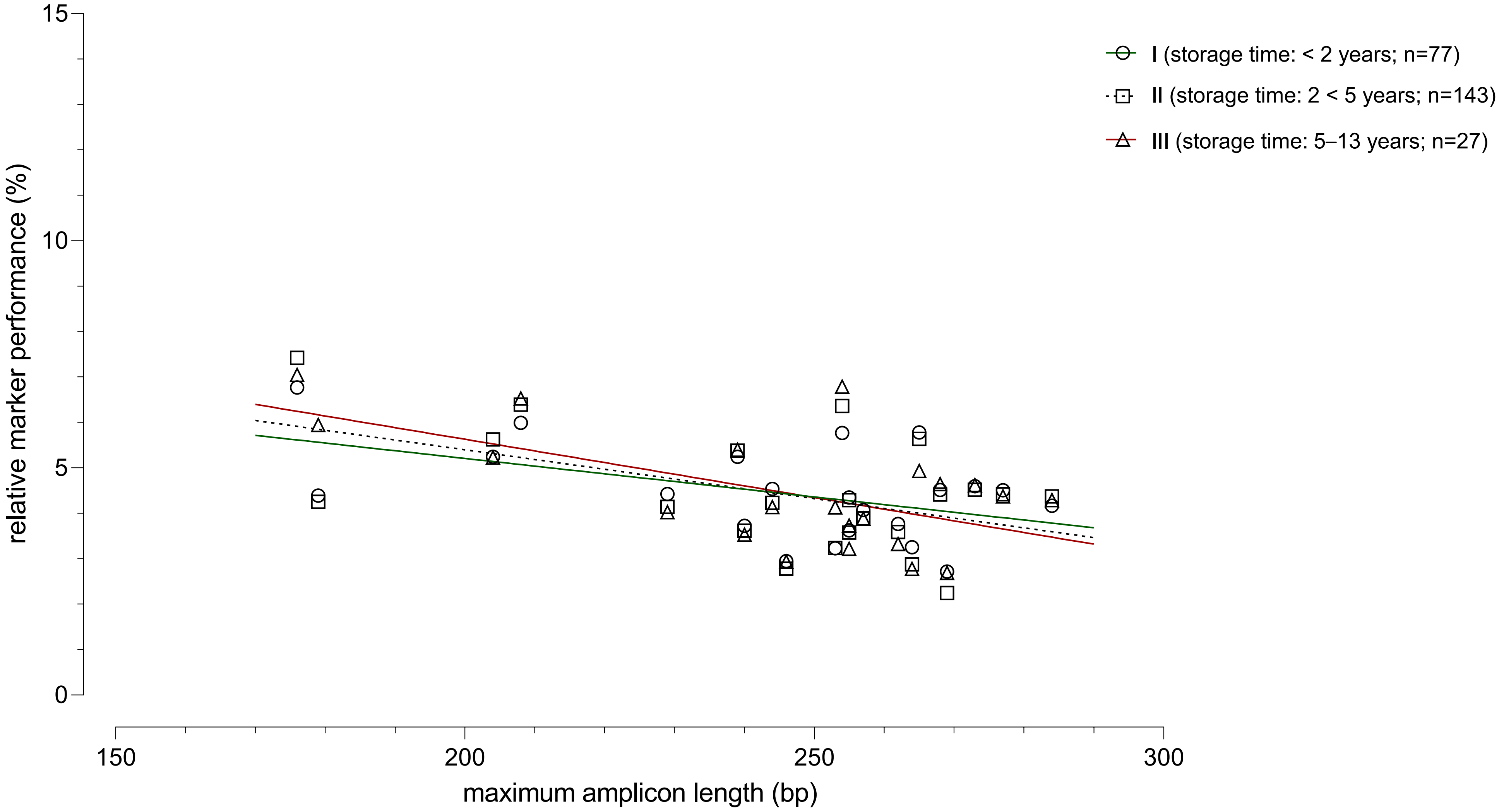

Fig. S2 Linear regression analysis for the relative marker performance depending on the maximum amplicon length. No indication for differences in the relative marker performance was observed between storage groups I–III, with the exception of TPOX. STR markers are shown in decreasing order of maximum amplicon length from left to right: D1S1656, TPOX, D2S441, D2S1338, D3S1358, FGA, D5S818, CSF1PO, D7S820, D8S1179, D10S1248, TH01, vWA, D12S391, D13S317, Penta E, D16S539, D18S51, D19S433, D21S11, Penta D, and D22S1045.
