# Supplementary material for: Exploring STR sequencing for forensic DNA intelligence databasing using the Austrian National DNA Database as an example
Source: Int J Legal Med. 2021 Aug 26;135(6):2235–46. doi: 10.1007/s00414-021-02685-x (PMC8523457; doi:10.1007/s00414-021-02685-x)
Supplement: Supplementary file 3 — Supplementary file3 (PDF 96 KB) [file 414_2021_2685_MOESM3_ESM.pdf]

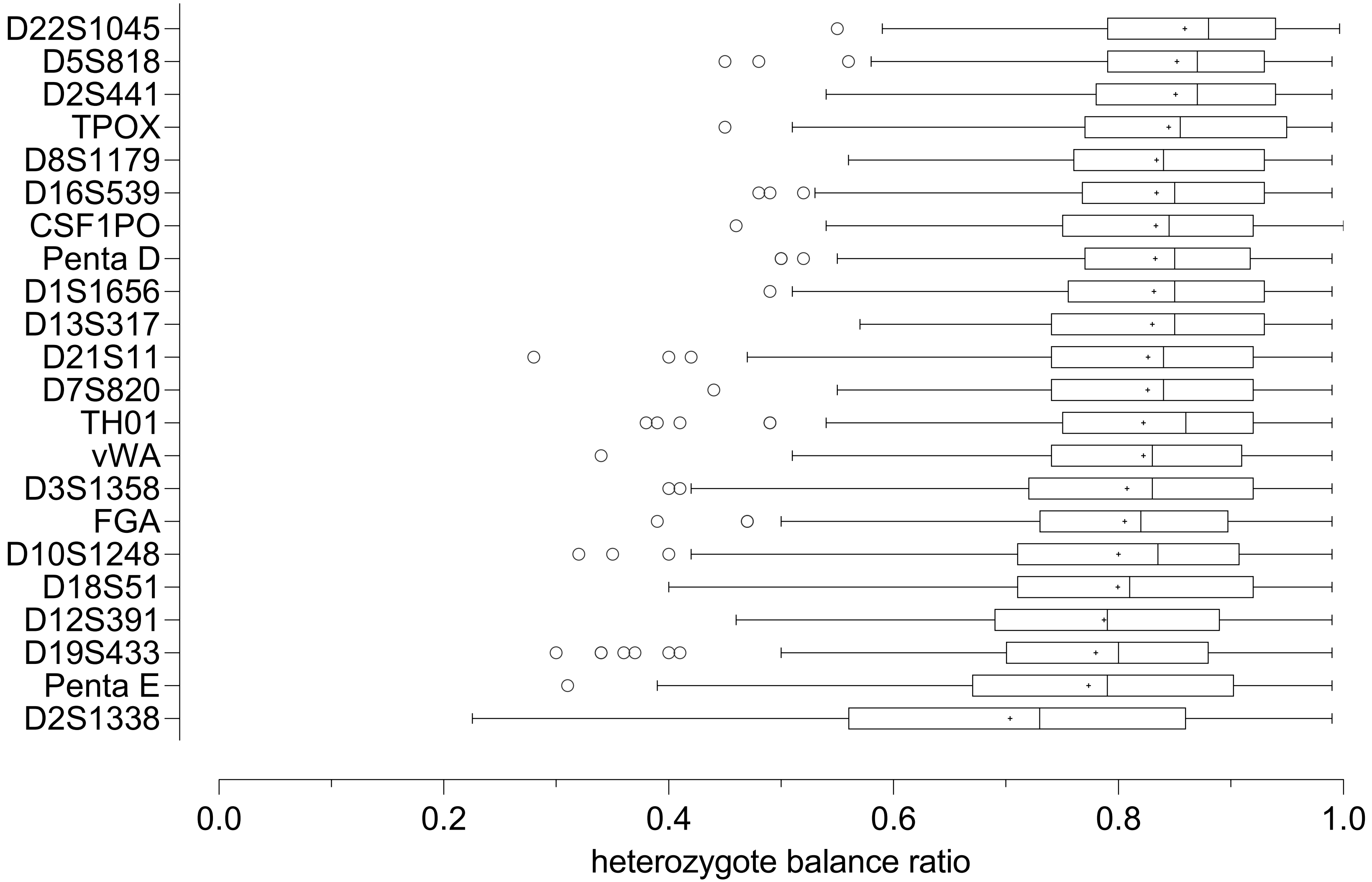


**Fig. S3** Box-whisker plot showing heterozygote balance (HB) ratios for 22 autosomal STRs. All STR markers showed average HB ratios ≥ 0.80 (mean shown as '+'), except D2S1338, Penta E, D19S433 and D12S391.
