# Supplementary material for: Exploring STR sequencing for forensic DNA intelligence databasing using the Austrian National DNA Database as an example
Source: Int J Legal Med. 2021 Aug 26;135(6):2235–46. doi: 10.1007/s00414-021-02685-x (PMC8523457; doi:10.1007/s00414-021-02685-x)
Supplement: Supplementary file 4 — Supplementary file4 (PDF 175 KB) [file 414_2021_2685_MOESM4_ESM.pdf]

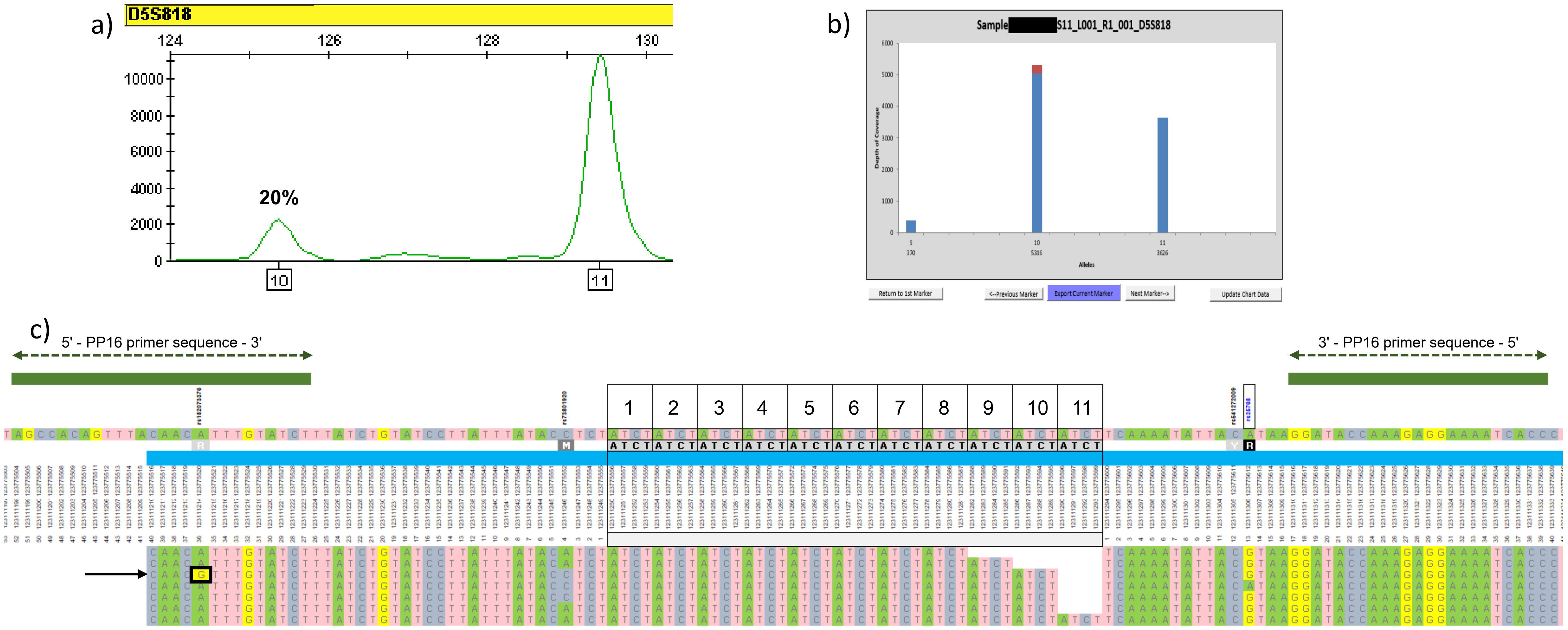

Fig. S4 Intra-locus imbalance at D5S818. During size-based STR analysis intra-locus imbalance was observed at D5S818 due to sequence variation located in close proximity of the 3' end of the PowerPlex 16 kit forward primer. The black arrow denotes the sequence string containing the A>G transition. This particular SNP (rs182073376) affects the PCR by decreasing thermal stability of the primer-template complex and reducing the PCR performance. **a)** CE electropherogram of D5S818 after amplification with the PowePlex 16 kit, **b)** MPS-based genotype result, **c)** aligned sequences showing different allele calls at D5S818.

Hölzl-Müller P., Bodner M., Berger B., and Parson W. "Exploring STR Sequencing for Forensic DNA Intelligence Databasing using the Austrian National DNA Database as an Example"
