# Supplementary material for: Exploring STR sequencing for forensic DNA intelligence databasing using the Austrian National DNA Database as an example
Source: Int J Legal Med. 2021 Aug 26;135(6):2235–46. doi: 10.1007/s00414-021-02685-x (PMC8523457; doi:10.1007/s00414-021-02685-x)
Supplement: Supplementary file 5 — Supplementary file5 (PDF 174 KB) [file 414_2021_2685_MOESM5_ESM.pdf]

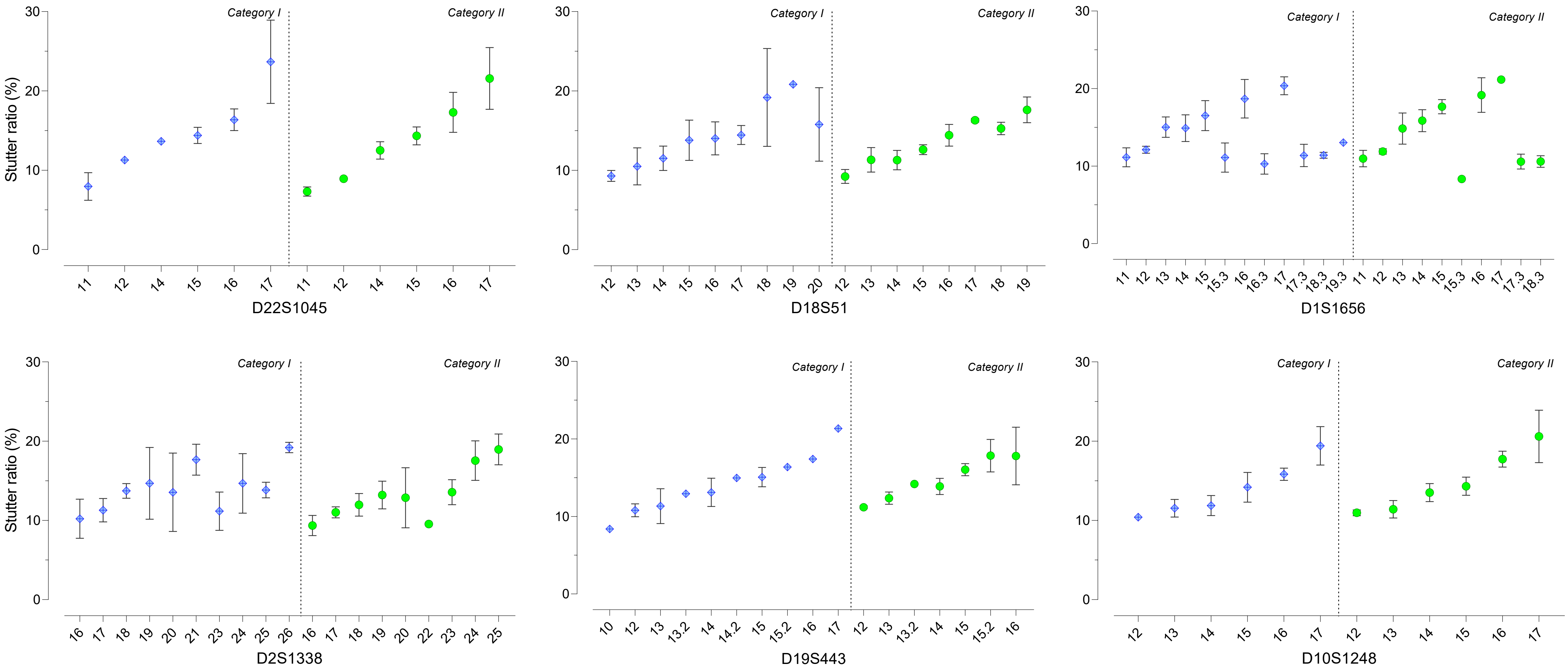

Fig. S5 Stutter analysis was performed on a subset of 50 samples that were selected according to the total number of reads (selection criteria: category I (blue rhombuses): ≤ 63,500 reads; category II (green circles): ≥ 199,000 reads). Mean stutter ratios in percent are shown for all STR markers (error bars denote stutter ranges). The x-axis of all figures relate to marker specific alleles, e.g. "D22S1045 alleles". Allele calls without symbol indicate alleles without detectable stutter products.

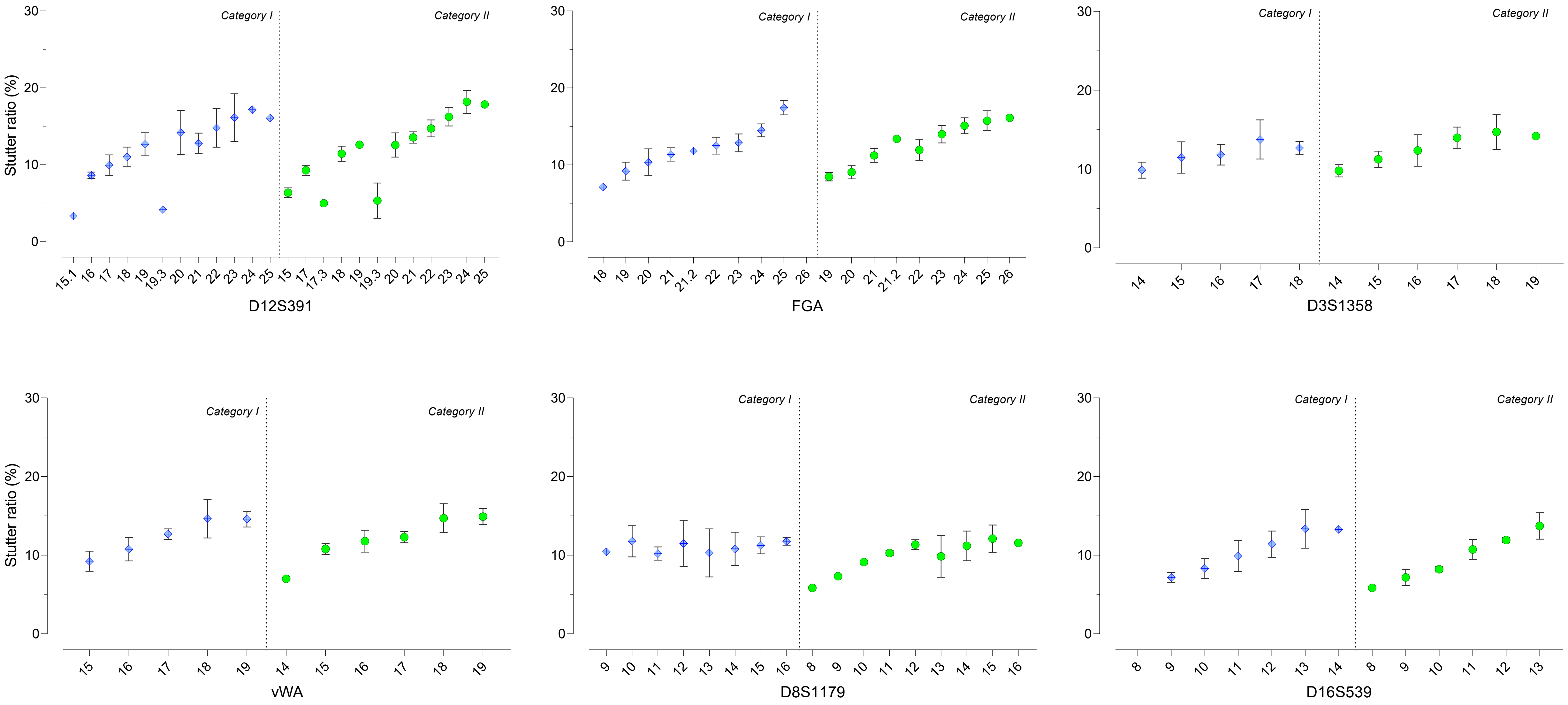
Stutter ratio (%)
Category I
Category II
D12S391
FGA
D3S1358
vWA
D8S1179
D16S539

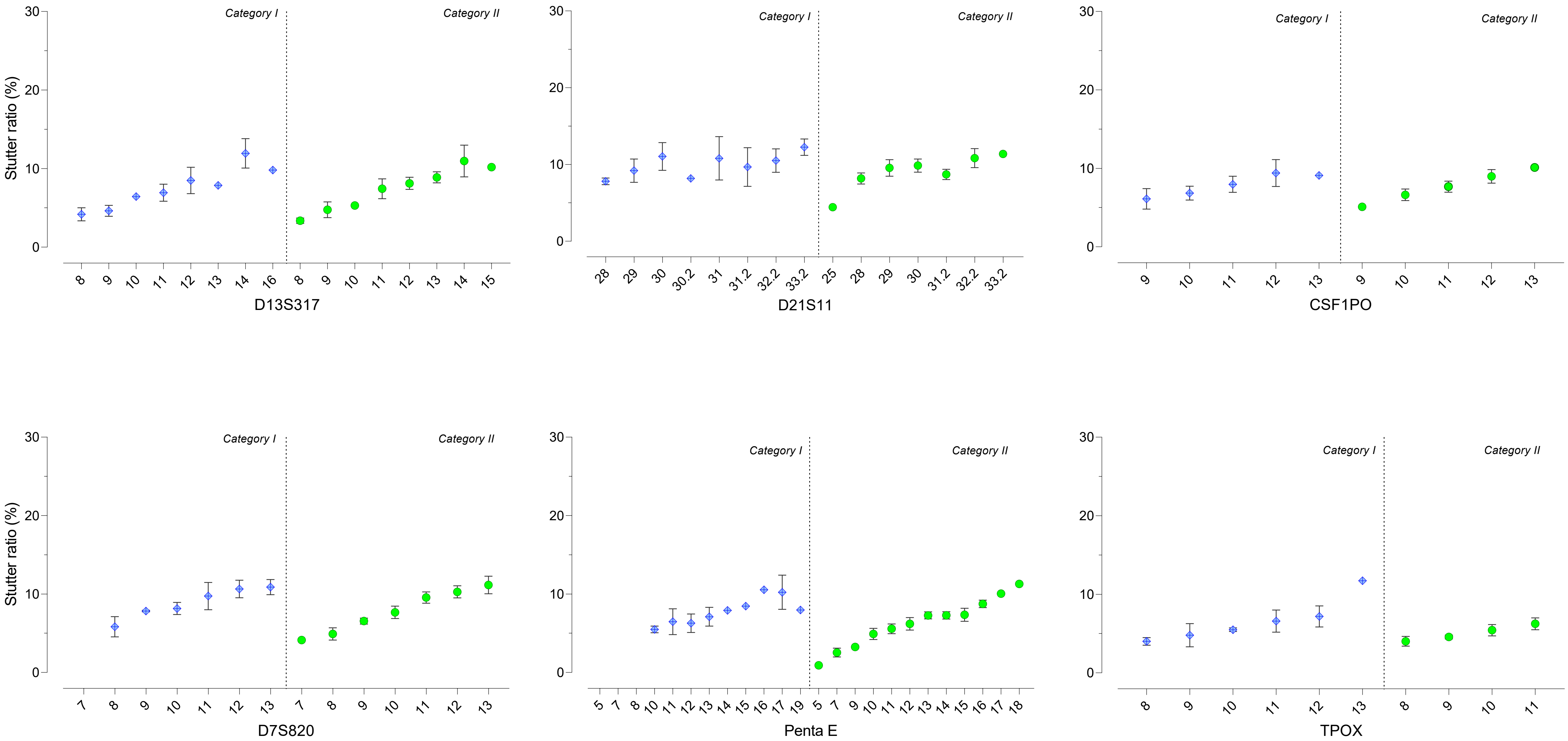
Stutter ratio (%)
30
20
10
0
Category I
Category II
8 9 10 11 12 13 14 16 8 9 10 11 12 13 14 15
D13S317
28 29 30 30.2 31 31.2 32.2 33.2 25 28 29 30 31.2 32.2 33.2
D21S11
9 10 11 12 13 9 10 11 12 13
CSF1PO
7 8 9 10 11 12 13 7 8 9 10 11 12 13
D7S820
5 7 8 10 11 12 13 14 15 16 17 19 5 7 9 10 11 12 13 14 15 16 17 18
Penta E
8 9 10 11 12 13 8 9 10 11
TPOX

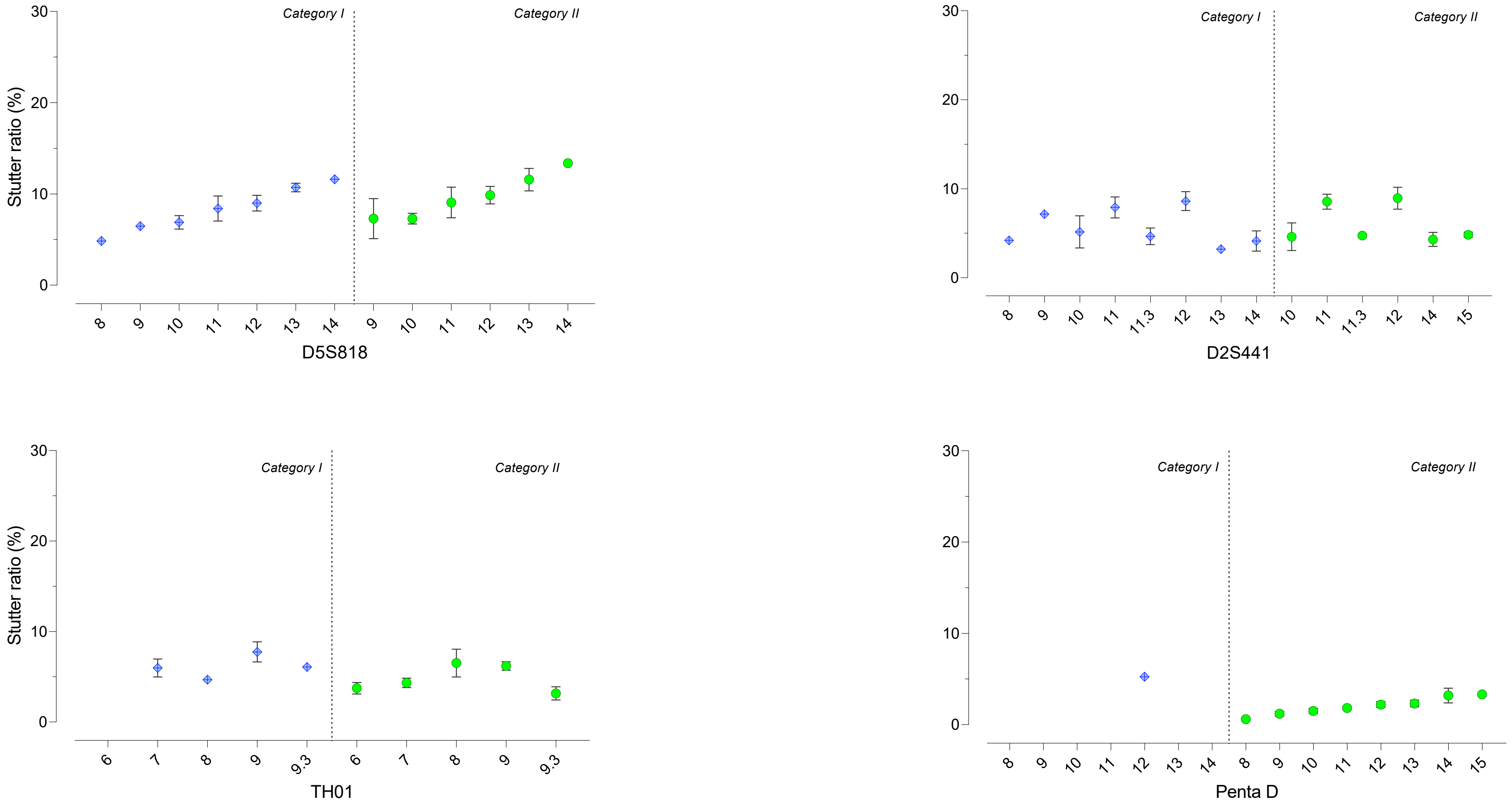
Stutter ratio (%)
0
10
20
30
Category I
Category II
8 9 10 11 12 13 14 9 10 11 12 13 14
D5S818
8 9 10 11 11.3 12 13 14 10 11 11.3 12 14 15
D2S441
6 7 8 9 9.3 6 7 8 9 9.3
TH01
8 9 10 11 12 13 14 8 9 10 11 12 13 14 15
Penta D
