# Supplementary material for: Exploring STR sequencing for forensic DNA intelligence databasing using the Austrian National DNA Database as an example
Source: Int J Legal Med. 2021 Aug 26;135(6):2235–46. doi: 10.1007/s00414-021-02685-x (PMC8523457; doi:10.1007/s00414-021-02685-x)
Supplement: Supplementary file 6 — Supplementary file6 (PDF 138 KB) [file 414_2021_2685_MOESM6_ESM.pdf]

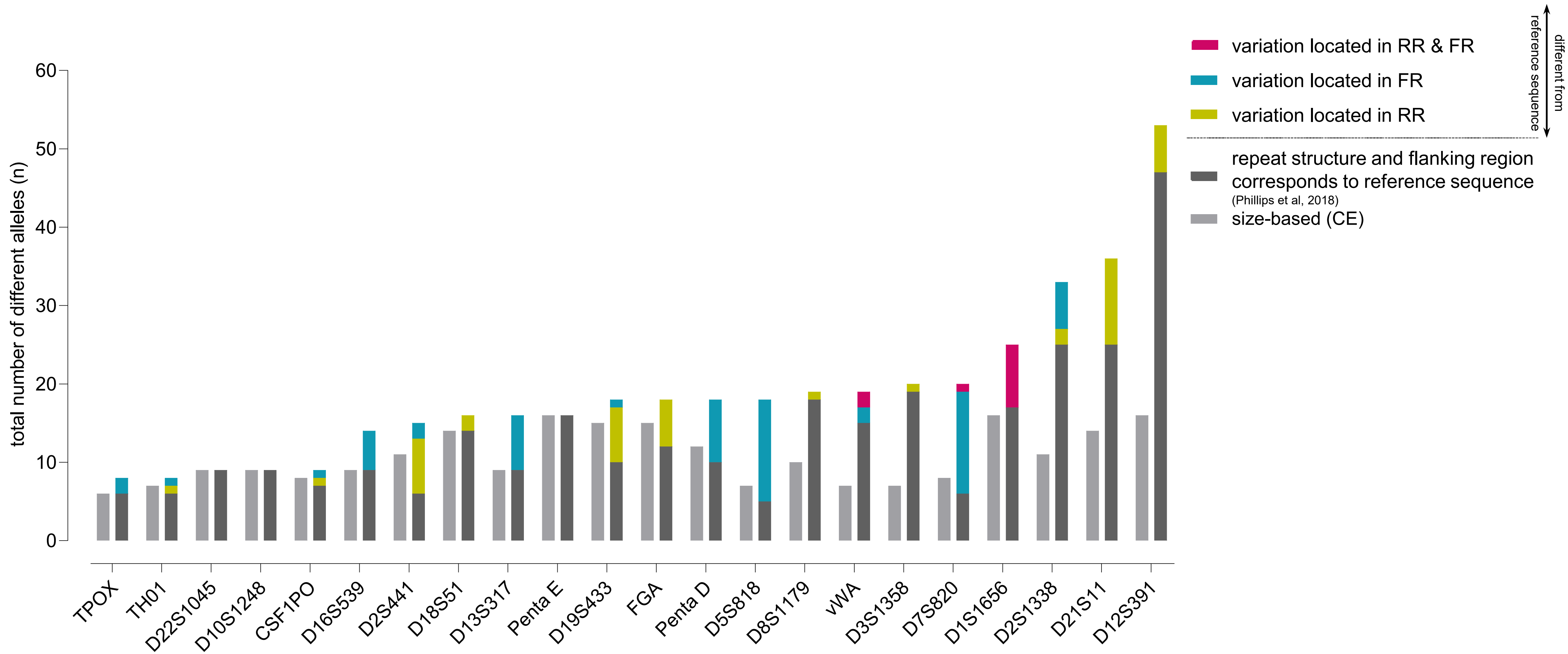

Fig. S6 The bar chart shows allelic variation in terms of the total number of distinguishable alleles using size-based and sequence-based STR analysis technologies. For CE data we counted the maximum number of discriminable alleles (light grey bars). MPS data were aligned to the corresponding reference sequence and categorized according to the position of the sequence variation using the updated Forensic STR Sequence Structure Guide v5 (Phillips et al, 2018) as template. Dark grey bars: repeat structures and flanking regions (FR) that correspond to the marker specific reference sequence. Olive yellow bars: repeat region (RR) variation includes any changes that affect the RR in relation to the reference sequence. Cyan bars: FR variation includes any changes that affect the FR in relation to the reference sequence. Pink bars: RR & FR variation includes changes that affect the RR & FR of the sequence string in relation to the reference sequence. Based on sequence information, MPS genotyping increased the detection of genetic variation for more than 86% of the included autosomal STRs, except for D10S1248, Penta E and D22S1045 (19 out of 22 markers).

Hölzl-Müller P., Bodner M., Berger B., and Parson W. "Exploring STR Sequencing for Forensic DNA Intelligence Databasing using the Austrian National DNA Database as an Example"
