# Supplementary material for: Exploring STR sequencing for forensic DNA intelligence databasing using the Austrian National DNA Database as an example
Source: Int J Legal Med. 2021 Aug 26;135(6):2235–46. doi: 10.1007/s00414-021-02685-x (PMC8523457; doi:10.1007/s00414-021-02685-x)
Supplement: Supplementary file 7 — Supplementary file7 (PDF 122 KB) [file 414_2021_2685_MOESM7_ESM.pdf]

a)

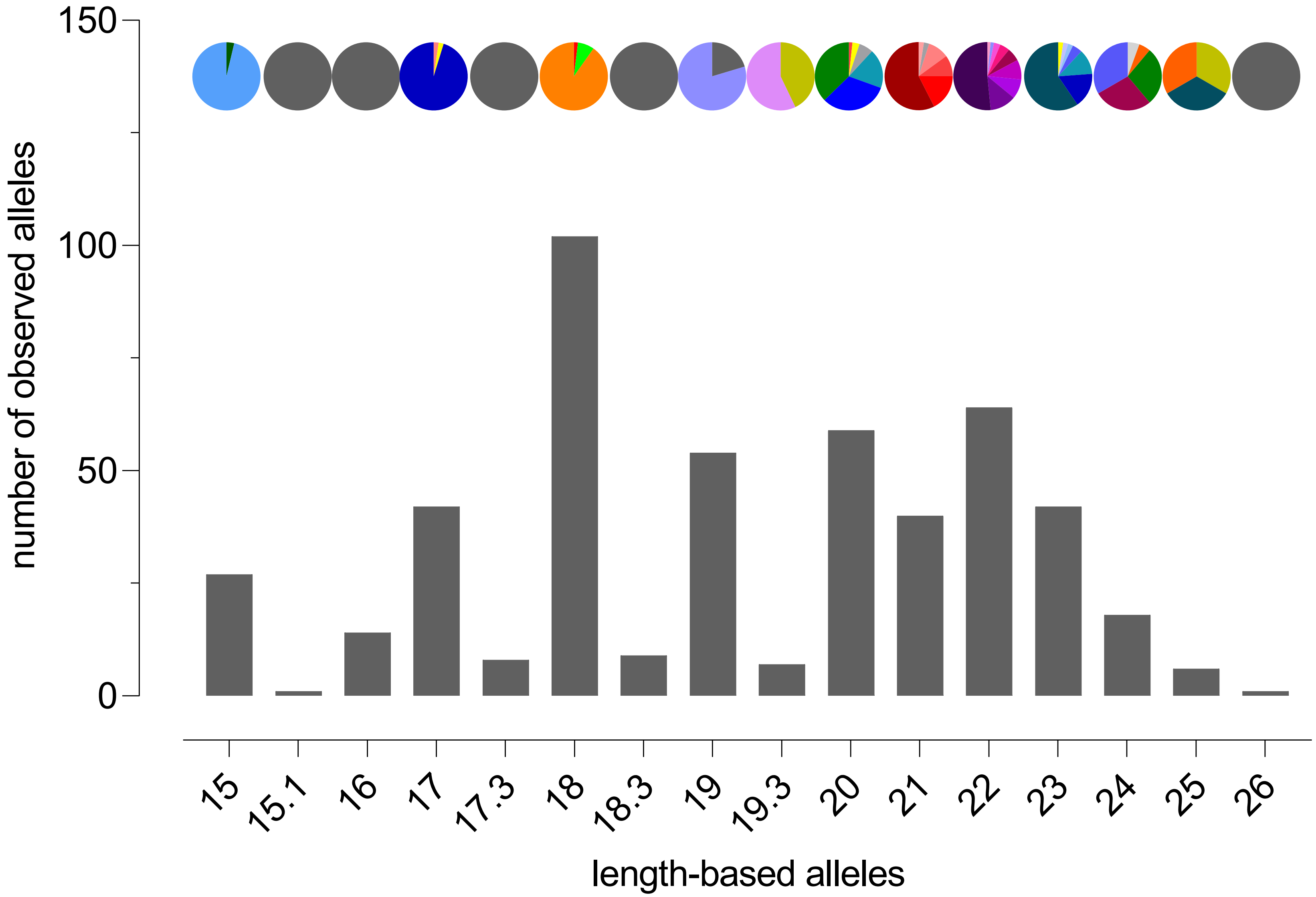

Fig. S7 Within the present dataset, including 22 autosomal STRs, particularly **a)** D12S391, **b)** D2S1338 and **c)** D21S11 were found to be highly variable. Compared to length-based STR analysis, MPS resulted in 3.3-fold, 3.0-fold and 2.6-fold increase in the number of distinguishable alleles, respectively. Pie charts on top of each bar represent the number (expressed in different colors) and relative distribution of observed sequence variants found in each length-based allele.

b)

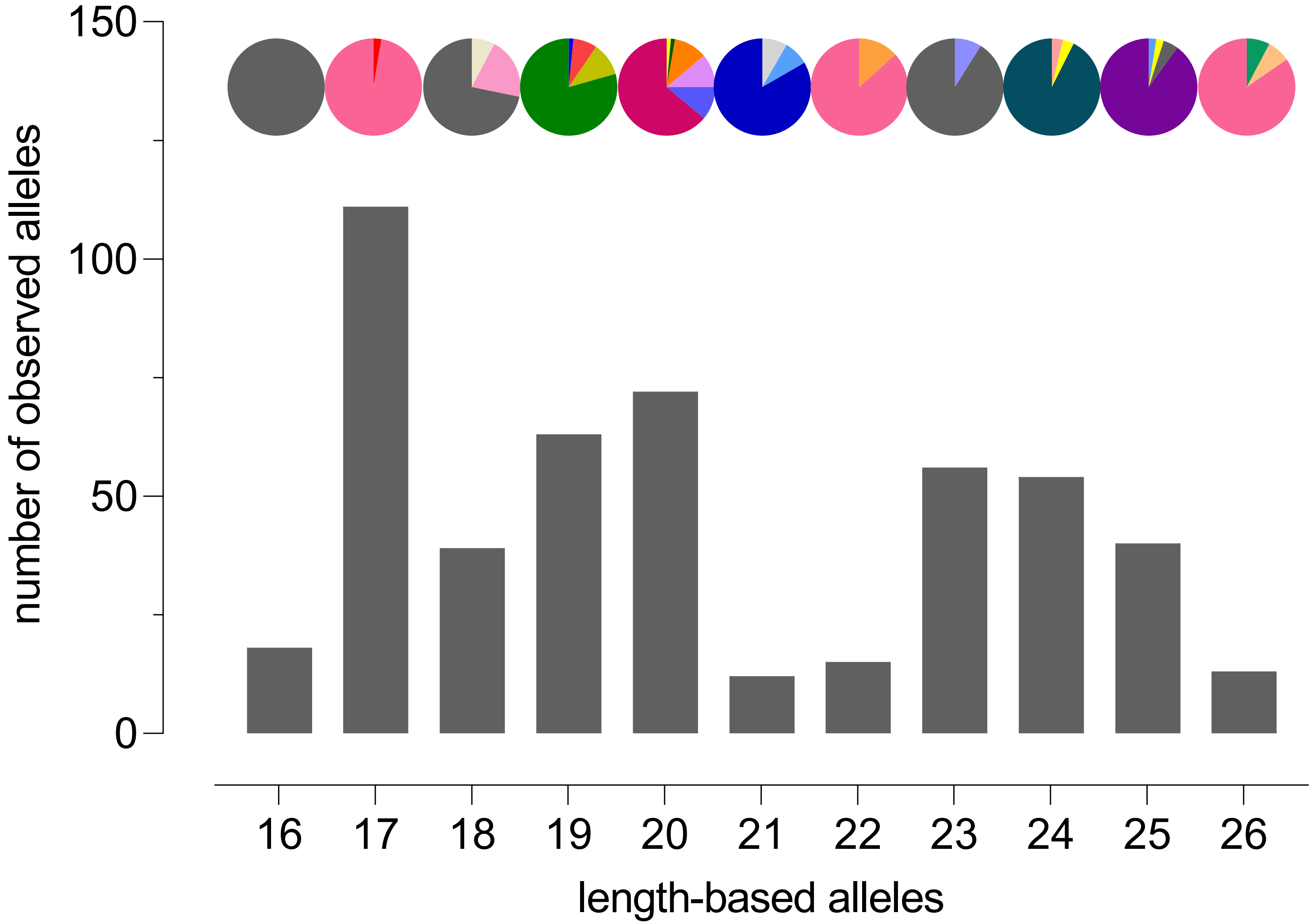
150
100
50
0
number of observed alleles
16
17
18
19
20
21
22
23
24
25
26
length-based alleles

c)

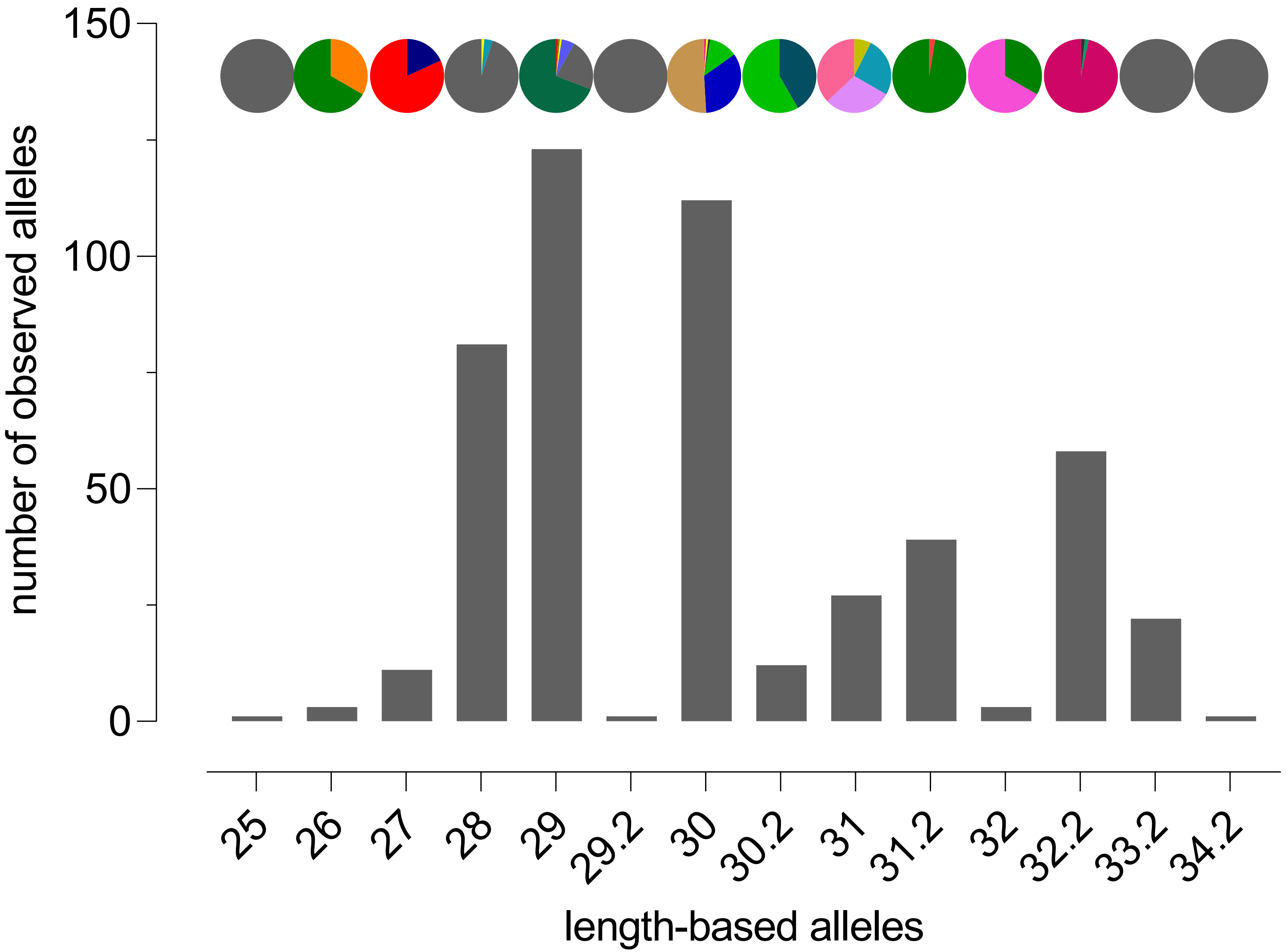

150
100
50
0
number of observed alleles
25
26
27
28
29
29.2
30
30.2
31
31.2
32
32.2
33.2
34.2
length-based alleles
